# Health professionals’ job satisfaction and associated factors at public health centers in West Ethiopia

**DOI:** 10.1186/s12960-017-0206-3

**Published:** 2017-05-30

**Authors:** Beyazin Kebede Deriba, Shimele Ololo Sinke, Berhane Megersa Ereso, Abebe Sorsa Badacho

**Affiliations:** 1 Horro Guduru Zone Health Department, Oromia Health Bureau, Horro, Ethiopia; 2 Department of Health Services Management, College of Public Health and Medicine, Jimma University, Po. box 378, Jimma, Ethiopia; 3 School of Public Health, College of Health Sciences and Medicine, Wolaita Sodo University, Sodo, Ethiopia

**Keywords:** Health professionals, Job satisfaction, Satisfaction score, Oromia, Ethiopia

## Abstract

**Background:**

Human resources are vital for delivering health services, and health systems cannot function effectively without sufficient numbers of skilled, motivated, and well-supported health workers. Job satisfaction of health workers is important for motivation and efficiency, as higher job satisfaction improves both employee performance and patient satisfaction. Even though several studies have addressed job satisfaction among healthcare professionals in different part of the world, there are relatively few studies on healthcare professionals’ job satisfaction in Ethiopia.

**Methods:**

A facility-based cross-sectional study was conducted among health professionals working in health centers in April 2015 using self-administered structured questionnaires. All 322 health professionals working in 23 randomly selected public health centers were included. Factor scores were computed for the identified items by varimax rotation to represent satisfaction. Multivariate linear regression analysis was performed, and the effect of independent variables on the regression factor score quantified.

**Results:**

Three hundred eight respondents participated with a response rate of 95.56%. The overall level of job satisfaction was 41.46%. Compensation (benefits) (beta 0.448 [95% CI 0.341 to 0.554]), recognition by management (beta 0.132 [95% CI 0.035 to 0.228]), and opportunity for development (beta 0.123 [95% CI 0.020 to 0.226]) were associated with job satisfaction. A unit increase in salary and incentives and recognition by management scores resulted in 0.459 (95% CI 0.356 to 0.561) and 0.156 (95% CI 0.065 to 0.247) unit increases in job satisfaction scores, respectively.

**Conclusions:**

The overall level of job satisfaction in health professionals was low. Salary and incentives, recognition by management, developmental opportunities, and patient appreciation were strong predictors of job satisfaction.

**Electronic supplementary material:**

The online version of this article (doi:10.1186/s12960-017-0206-3) contains supplementary material, which is available to authorized users.

## Background

Health services are affected by many factors such as human resources, health delivery, and health infrastructures. Human resources are a vital component for delivering health services, and health systems cannot function effectively without sufficient numbers of skilled, motivated, and supported health workers [1]. Developing capable, motivated, and supported health workers is essential to overcome bottlenecks in achieving national and global health goals. The work force is central to advancing health in every health system [2].

There should be an optimal number and professional mix of human resources to deliver effective coverage and quality of the intended services. Despite health and poverty eradication being high on the international agenda, with significant achievements made in some developing countries, progress remains extremely slow in Africa. This is primarily due to weak health systems characterized by severe shortages, poor capacity, and de-motivated health workers at all levels across the continent [3, 4].

The presence of high-quality and motivated staff is not only a key aspect of health system performance but also one of the most difficult inputs to ensure [5]. Job satisfaction of health workers is important for motivating employees and improving efficiency, as higher job satisfaction is known to improve employee performance and patient satisfaction. Conversely, job dissatisfaction results in burn out, and high employee turnover exacerbates current shortages and results in serious under staffing of healthcare facilities [6].

The definition of job satisfaction varies from person to person and even in the same person from time to time. Job satisfaction generally refers to an evaluation made by the employee of the job and its environment [7]. Job satisfaction can be quantified as the difference between the amount of rewards workers receive and the amount they believe they should receive [8].

Several studies from Africa have shown that job dissatisfaction results from one or more attributes of the work environment such as poor living and working conditions, problems with leadership, inadequate equipment and supplies, lack of recognition for good work, stress due to heavy workloads, and limited opportunities for career development and advancement [9, 10].

Ethiopia, as with many other sub-Saharan African countries, also faces shortages in skilled health professionals. This has been accelerated by a variety of factors and has resulted in health worker migration from the public sector, geographical imbalances in workforce staffing, and increasing attrition rates. This health workforce shortage, failures in employing professionals at the right time, retaining them, managing them, and budget shortages with irregular continuing education have critically affected the Ethiopian health system [11].

The loss of clinical staff from low- and middle-income countries (LMICs) is crippling already fragile healthcare systems. Health worker retention is critical for health system performance, and a key problem is how best to motivate and retain health workers [12]. A study conducted on brain drain and retention of health professionals in Africa showed that the continent faces a health crisis due to the very low funding of health services and the deterioration of health service infrastructure. These factors threaten the performance of health workers and job satisfaction [10].

As well as the positive effects of job satisfaction, job dissatisfaction has a negative impact on the organization by decreasing productivity and increasing work accidents, intra-organizational conflict, employee turnover, tardiness, and grievances [13]. Although several reforms and policies have been developed to address health problems in Africa, little attention has been given to health worker job satisfaction and their motivation [14].

In Ethiopia, despite the fact that the government is making substantial programs toward increasing the number, category, and quality of health workers and health infrastructure, service programs still face problems. The national health services tend not to attract health workers since little attention is given to provide incentives, motivation, and adequate remuneration. Thus, effort needs to be made to develop and maintain health worker job satisfaction after identification of significant influencing factors [15].

According to the World Bank 2010 study, health workers tend to be unsatisfied with most aspects of their job in Ethiopia and especially their salary, training opportunities, and chances of promotion. Eighty percent of health professionals are either “unsatisfied” or “very unsatisfied” with their current salary [16].

Although there have been many studies worldwide to address job satisfaction and its factors in health professionals working in hospitals, most previous studies have concentrated on specific profession categories such as nurses, pharmacists, or doctors [13, 17, 18]. Few studies have investigated job satisfaction in different healthcare professionals in Ethiopia, and those that have do not include health center structure. Therefore, this study attempted to address this gap to identify factors influencing job satisfaction among health workers and the extent to which these problems exist in health centers in West Ethiopia.

## Methods

### Study area and period

The study was conducted at selected public health centers in Horro Guduru Wollega, Oromia Regional State, West Ethiopia, from April 1–30, 2015. Horro Guduru Wollega Zone is one of the 18 zones of Oromia Regional State. Shambu, the capital of the zone, is located 314 km west of Addis Ababa. The total estimated population of the zone is 661 790.

The zone has 10 districts, 9 rural and 1 town administration with 46 health centers and 1 zonal hospital. Five hundred seventy health professionals are employed in the 46 health centers of the zone: 288 nurses, 82 pharmacists, 68 midwives, 32 health officers, 88 laboratory professionals, and 12 environmental health professionals.

### Study design

This was a facility-based cross-sectional study.

### Sample size and sampling technique

Twenty-three health centers were randomly selected from the 46 health centers. All 322 health professionals in these 23 health centers were included in the study.

### Data collection tools

A structured, self-administered questionnaire was used to collect data from participants (Additional file 1). The questionnaire was prepared in English and translated to the local language Afan Oromo and back to English to check for consistency. The study variables were adopted by reviewing relevant literature.

Data collectors were diploma holders fluent in the local language. The data collectors were given 3-day training on the study objectives, the method of data collection, and the tools for data collection. The data collection tool was pre-tested in another study area.

### Data analysis

Data were entered into EpiData version 3.1 for double verification and exported to SPSS version 20.0 for further analysis. The frequency distribution of all the variables was examined to check for data entry errors. Each study was described using descriptive statistics. Factor analysis was employed for Likert scale instruments to extract factor(s) representing each of the scales and to obtain factor scores. To determine the reliability of the questionnaire, the Cronbach’s alpha internal consistency test was used for each dimension. Cronbach’s alpha values greater than 0.7 were regarded as acceptable and those ≥0.7 were subjected to factor/principal component analysis (PCA). The Kaiser-Meyer-Olkin test and Bartlett’s test were used to assess the appropriateness of using factor analysis and to identify job satisfaction factors.

Factors with eigenvalues greater than one were considered in subsequent analyses. Factor scores were computed for the item identified to represent the satisfaction scale by varimax rotation. Multivariate linear regression analysis was performed, and the effect of independent variables on the regression factor score of the dependent variable quantified. Explanatory variables that had a statistically significant association with the dependent variable (*p* < 0.05) were entered into the final regression model.

### Measurement

Job satisfaction refers to the perception of health professionals about their jobs. It was measured by six items in the satisfaction questionnaire on a 5-point Likert scale from strongly disagree (1) to strongly agree (5) (maximum score 30, minimum score 6). PCA was conducted, and the component score was used as the dependent variable for further analysis. Satisfaction levels were calculated based on the percentage of maximum scale scores. The percentage of maximum scale scores was computed using the formula

$$ \begin{array}{l}\mathrm{Percentage}\ \mathrm{mean}\ \mathrm{score} = \kern0.5em \left(\underset{\bar{\mkern6mu}}{\mathrm{actual}\ \mathrm{score}-\mathrm{potential}\ \mathrm{minimum}\ \mathrm{score}}\right)\times 100\%\\ {}\kern7.76em \left(\mathrm{potential}\  \max .\ \mathrm{score}-\mathrm{potential}\  \min .\ \mathrm{score}\right)\end{array} $$

The average of the individual level percentage scale scores and the overall level of satisfaction of the study population were taken [19].

Compensation and benefits refers to health professionals’ total pay and was measured by seven items on a 5-point Likert scale from strongly disagree (1) to strongly agree (5). The seven items yielded a maximum score of 35 and a minimum score of 7. PCA was conducted, which produced two meaningful factors with eigenvalues greater than one, which were renamed “salary (incentives)” and “benefit packages.” These two factors accounted for about 68% of the total variance and were used for further analysis.

Recognition and reward refers to acknowledging and rewarding employee performance to encourage effort and was measured by seven items on a 5-point Likert scale from strongly disagree (1) to strongly agree (5) to give a maximum score of 35 and a minimum score of 7. PCA was conducted to produce two meaningful factors with eigenvalues greater than one, which were renamed “recognition by management” and “patient appreciation.” These two factors accounted for about 62% of the total variance.

Working environment involves the physical, geographical location as well as the immediate surroundings of the work place and infrastructure of the health center including availability of essential materials and supplies. This was measured with eight items on a 5-point Likert scale from strongly disagree (1) to strongly agree (5). PCA was conducted that produced one meaningful factor with an eigenvalue greater than one that accounted for about 50% of the total variance. The component score was used in further analyses.

Developmental opportunities refers to the perception of the likelihood of being promoted by the health center and was measured by five items on a 5-point Likert scale from strongly disagree (1) to strongly agree (5). These five items yielded a maximum score of 25 and a minimum score of 5. To examine the underlying factors (components) of the developmental opportunity scale, PCA was conducted, which produced one meaningful factor with an eigenvalue greater than one accounting for about 67% of the total variance.

Relationship with management refers to the perception of health professionals toward their health center head and management bodies. Twelve items on a 5-point Likert scale were used. All 12 items yielded a maximum score of 60 and a minimum score of 12. PCA was conducted that produced two meaningful factors with eigenvalues greater than one that were renamed “better management” and “good communication.” These accounted for about 67% of the total variance.

Staff working relationship refers to the working relationship within staff and was measured by four items (maximum score 20, minimum score 4). To examine the underlying factors (components) of the staff working relationship scale, PCA was conducted. The factor staff working relationship accounted for about 72% of the total variance, and the component score was used for further analysis.

## Results

### Socio-demographic characteristics of the respondents

Three hundred twenty two survey questionnaires were distributed, of which 320 were returned. From the returned questionnaires, 308 were completed fully to give a response rate of 95.65%.

One hundred ninety three (62.7%) respondents were males. The mean age was 27.0 (±4.35) years with a range of 20 to 45 years. One hundred seventy one (55.5%) of the respondents were single, and 246 (79.9%) were diploma holders. Moreover, 289 (93.8%) respondents were Oromo by ethnicity, and 192 (62.3%) were protestant. Two hundred twenty one (71.8%) had duration of service less than 5 years in the health centers. Fifty-one percent of the respondents were nurses. Eighty-three (26.9%) had a monthly income of 1300–1500 Ethiopian birr (65–75 US dollars) (Table 1).

**Table 1:** Socio-demographic characteristics of health professionals working in Horro Guduru Wollega public health centers in West Ethiopia (*n* = 308)

Variables		Number	Percent
Sex	Male	193	62.7
	Female	115	37.3
Age category	<30 years	260	84.4
	≥30 years	48	15.6
Level of education	Diploma	246	79.9
	Degree	62	20.1
Marital status	Single	171	55.5
	Married	135	43.8
	Others	2	0.7
Religion	Protestant	192	62.3
	Orthodox	92	6.5
	Muslim	92	29.9
	Catholic	4	1.3
Ethnicity	Oromo	289	93.8
	Amhara	19	6.1
Profession	Health officer	18	5.8
	Nurse	157	51.0
	Midwife	40	13.0
	Lab	46	14.9
	Pharmacy	42	13.6
	Others	5	1.6
Working experience	1–5 years	221	71.8
	6–10 years	66	21.4
	>10 years	21	6.8
Monthly income	<1300	80	26.0
	1300–1500	83	26.9
	1501–1969	71	23.1
	>1970	74	24

### Description of health professionals’ general job satisfaction

Of the 308 respondents, almost half (151, 49%) agreed or strongly agreed that their job had more advantages than disadvantages. However, over two thirds (216, 70.2%) of the respondents disagreed or strongly disagreed that their income properly reflected their work. Regarding performance evaluation, the majority of respondents (104, 33.8% and 97, 31.5%) disagreed or strongly disagreed, respectively.

### Level of job satisfaction

The overall level of health professionals’ job satisfaction was 41.46%.

### Socio-demographic predictors of health professionals’ job satisfaction score

The relationship between socio-demographic variables and job satisfaction score was quantified. Socio-demographic variables explained only 3.8% of the variability in job satisfaction score. Accordingly, education status and monthly income were significantly associated with job satisfaction score in bivariate analysis. Respondents who were degree holders had a 0.375 unit greater job satisfaction score than diploma holders (95% CI, 0.098 to 0.652). Similarly, respondents who had a monthly salary of >1970 Ethiopian birr (98.5 US dollars) had a 0.320 unit greater job satisfaction score than those with a monthly income of 1301–1500 Ethiopian birr (65–75 US dollars) (95% CI, 0.008 to 0.633).

### Job-related factors as predictors of health professionals’ job satisfaction score

Variables related to job satisfaction such as compensation and benefits, recognition and reward, working environment, developmental opportunities, relation with management, and staff working relationship were entered into the model. Accordingly, salary and incentives, benefit packages, recognition by management, patient appreciation, working environment, developmental opportunities, better management, clear communication, and staff working relationship showed statistically significant associations with job satisfaction scores. All of the variables had positive associations with job satisfaction scores. A unit increase in salary and incentives score caused an increment of 0.608 units in job satisfaction score (95% CI, 0.519 to 0.697). Similarly, a unit increase in developmental opportunities and patient appreciation score produced 0.447 (95% CI, 0.346 to 0.548) and 0.365 (95% CI, 0.260 to 0.470) unit increases in job satisfaction scores, respectively (Table 2).

**Table 2:** Association of job-related factors and health professionals’ job satisfaction score in Horro Guduru Wollega public health centers in West Ethiopia

Variables	Unstandardized coefficient		Standardized coefficient	*T*	*p* value	Confidence interval	
	Beta	Std. error	Beta			LB	UB
Enough salary and incentives	0.608	0.045	0.608	13.392	0.000	0.519	0.697
Benefit package	0.108	0.057	0.108	1.908	0.057	−0.003	0.220
Recognition by management	0.296	0.055	0.296	5.424	0.000	0.189	0.404
Patient appreciation	0.365	0.053	0.365	6.860	0.000	0.260	0.470
Working environment	0.358	0.053	0.358	6.699	0.000	0.253	0.463
Developmental opportunities	0.447	0.051	0.447	8.744	0.000	0.346	0.548
Better management	0.319	0.054	0.319	5.889	0.000	0.212	0.426
Clear communication	0.252	0.055	0.252	5.453	0.000	0.143	0.361
Staff working relationship	0.271	0.055	0.271	4.934	0.000	0.163	0.380

### Predictors of job satisfaction scores

All of the variables with *p* values <0.25 with health professionals’ job satisfaction scores in bivariate linear regression analyses were entered into a multiple regression model. The final model explained about 44.9% of the variation in job satisfaction score. Salary and incentives, recognition by management, developmental opportunities, and patient appreciation were found to be strong predictors of job satisfaction score. Sufficient salary and incentives had the largest influence on the level of job satisfaction, but none of the demographic characteristics had a significant association with job satisfaction. A unit increase in salary and incentives score and recognition by management score caused 0.459 (95% CI, 0.356 to 0.561) and 0.156 (95% CI, 0.065 to 0.247) unit increases in job satisfaction scores, respectively (Table 3).

**Table 3:** Predictors of health professionals’ job satisfaction score in Horro Guduru Wollega Zone public health centers in West Ethiopia, April 2015

Variables	Unstandardized coefficient B		Standardized coefficient B	*t*	*p* value	95% CI for B	
	Beta	Std. error				LB	UB
Enough salary and incentives	0.459	0.052	0.459	8.783	0.000	0.356	0.561
Recognition by management	0.156	0.046	0.156	3.363	0.001	0.065	0.222
Patient appreciation	0.125	0.049	0.125	2.534	0.012	0.028	0.222
Developmental opportunities	0.123	0.052	0.123	2.348	0.020	0.020	0.226

## Discussion

This study was important to identify the factors that affect health professionals’ job satisfaction to focus attention on particular factors and provide possible interventions. Previous studies on health professionals’ job satisfaction in Ethiopia have been limited to the hospital setting and specific professions, making them difficult to directly compare with this study [20–22].

The overall level of health professionals’ job satisfaction was 41.46%, suggesting that most health professionals are not satisfied with their current job. This result was lower than that found in similar studies conducted in Egypt, Greece, and China, in which the overall level of job satisfaction was 61.24%, 74.63%, and 83.3%, respectively. This difference might be because the economic status and working conditions in these countries are better than those in Ethiopia [23–25]. However, this result was higher than a study of nurses in Addis Ababa and Pakistan, with overall levels of job satisfaction of 37% and 33%, respectively. This might be explained by the work load among nurses in these health facilities [22, 26].

No socio-demographic characteristic was significantly associated with job satisfaction score in the current study, consistent with similar studies conducted in health centers in Turkey and Pakistan [27, 28]. However, in a Malaysian study on human resources employees, gender and marital status did not affect job satisfaction but age, tenure, and education level did [29]. Therefore, previous studies are inconclusive and mixed, perhaps due to differences in culture and the nature of the employees studied.

We report that sufficient salary and incentives, recognition by management, patient appreciation, and developmental opportunities were significant predictors of job satisfaction. Salary (incentives) was the strongest predictor of job satisfaction (beta 0.448) followed by recognition by management (beta 0.132). This is consistent with studies from Uganda and Nigeria among health professionals, in which all of these variables were predictors of job satisfaction. There is likely to be some commonality in these developing countries since the working conditions and the health systems of these countries are likely to be similar [30, 31].

We also considered compensation (benefits); that is, financial and non-financial benefits from public health centers. Salary (incentives) was significant and entered into the final model. This is in line with a Ugandan study in which salary (incentives) was a significant predictor of the overall level of job satisfaction. Similarly, studies from Turkey and Malaysia also reported that salary was a strong predictor of overall job satisfaction. If an employee is compensated enough according to his needs, he is more likely to manage overload work. According to Herzberg’s hygiene theory, salary is a factor that leads employees from dissatisfaction to no dissatisfaction [27, 29, 32].

Recognition by management and patient appreciation were significantly associated with job satisfaction (*p* < 0.05). Recognition of staff can be one of the easiest and most cost-effective strategies to retain experienced health professionals. A study from the Sidama Zone public health facilities indicate that health workers who perceived having more recognition for outstanding performance or achievements reported a higher level of job satisfaction. This result was supported by Herzberg’s and Maslow’s theories, which identified recognition as a determinant of job satisfaction [21, 33].

Developmental opportunities was a dimension that also affected satisfaction, which consists of variables related to training, education, and fairness and chance of promotion. Our results were consistent with similar studies from Pakistan that reported that developmental opportunities was significantly associated with job satisfaction in the context of most respondents being dissatisfied with the professional and development opportunities received during their professional life. South African and Pakistani studies also reported that developmental opportunities was directly and positively related to job satisfaction [34, 35].

There was no significant association between job satisfaction and working environment or relationship with management, in contrast to studies from Malaysia. This may be because most health professionals were working at home and the institutional management was different [28, 29, 34].

### Limitation of the study

Although the participants were assured of confidentiality, there was a possibility that they either over- or under-reported their level of satisfaction.

## Conclusions

This study revealed that the level of health professionals’ job satisfaction was generally low. There was no significant association between socio-demographic variables and job satisfaction. Among the job-related factors, compensation (benefits), developmental opportunities, and recognition (award) significantly influences the job satisfaction of health professionals. There was no significant association between job satisfaction and staff working relationship, working environment, or relationship with management. Salary and incentives, recognition by management, developmental opportunities, and patient appreciation were strong predictors of health professionals’ job satisfaction.

## Additional file

Additional file 1: (SAV 55 kb)
